# Correction: Unstructured satellite survey detects up to 20% of archaeological sites in coastal valleys of southern Peru

**DOI:** 10.1371/journal.pone.0328409

**Published:** 2025-07-15

**Authors:** Thomas J. Snyder, Randall Haas

The two authors, Thomas J. Snyder and Randall Haas, should be noted as shared first co-corresponding authors on this work.
