# Supplementary material for: Unstructured satellite survey detects up to 20% of archaeological sites in coastal valleys of southern Peru
Source: PLoS One. 2024 Feb 6;19(2):e0292272. doi: 10.1371/journal.pone.0292272 (PMC10846729; doi:10.1371/journal.pone.0292272)
Supplement: S1 File — All R code used in the analysis of raw data. (DOCX) [file pone.0292272.s001.docx]

#### Satellite Survey Analysis Script #####

### R Statistical Computing Environment###

## Version 1.3.1903 ##

### Loads library packages

library(rgdal)

library(MASS)

library(sp)

library(lme4)

library(lmerTest)

library(raster)

##Determines buffer size (118)

p <- read.csv('’) # Imports random sample of sites

pn <- fitdistr(as.numeric(p$offset), 'lognormal') # fits offset to lognormal distribution

buffer <- qlnorm(.95, pn$estimate[1],pn$estimate[2])

##Creates spatial polygon from pedestrian data

x <- readOGR('') # Reads pedestrian survey OGR data

plot(x, pch = 1) # Plots points, visual data inspection

data.frame(x)

head(x)

plot(table(x$latest))

plot(table(x$earliest))

b <- buffer(x, width = 320, dissolve = FALSE)

plot(b)

## Loads in survey data

1 <- readOGR('')

11 <- readOGR('’)

7 <- readOGR('')

6 <- readOGR('')

9 <- readOGR('')

10 <- readOGR('')

3 <- readOGR('')

11 <- readOGR('')

8 <- readOGR('')

4 <- readOGR('')

5 <- readOGR('')

2 <- readOGR('')

##Checks overlap; points not in the polygon returns NA

1found <- over(1, b)

2found <- over(2, b)

3found <- over(3, b)

4found <- over(4, b)

6found <- over(6, b)

7found <- over(7, b)

8found <- over(8, b)

5found <- over(5, b)

9found <- over(9, b)

10found <- over(10, b)

11found <- over(11, b)

11found <- over(11, b)

11found

##Counts no. sites found

1true <- subset(1found, 3uid!='<NA>')

nrow(1true)

2true <- subset(2found, 3uid!='<NA>')

nrow(2true)

3true <- subset(3found, 3uid!='<NA>')

nrow(3true)

4true <- subset(4found, 3uid!='<NA>')

nrow(4true)

6true <- subset(6found, 3uid!='<NA>')

nrow(6true)

7true <- subset(7found, 3uid!='<NA>')

nrow(7true)

8true <- subset(8found, 3uid!='<NA>')

nrow(8true)

5true <- subset(5found, 3uid!='<NA>')

nrow(5true)

9true <- subset(9found, 3uid!='<NA>')

nrow(9true)

11true <- subset(11found, 3uid!='<NA>')

nrow(11true)

10true <- subset(10found, 3uid!='<NA>')

nrow(10true)

11true <- subset(11found, 3uid!='<NA>')

nrow(11true)

t.total <- c(nrow(1true),

nrow(3true),

nrow(2true),

nrow(4true),

nrow(5true),

nrow(6true),

nrow(7true),

nrow(8true),

nrow(9true),

nrow(10true),

nrow(11true),

nrow(11true))

##Compares to pedestrian survey

t.frac <- c(nrow(1true)/546,

nrow(2true)/546,

nrow(3true)/546,

nrow(4true)/546,

nrow(5true)/546,

nrow(6true)/546,

nrow(7true)/546,

nrow(8true)/546,

nrow(9true)/546,

nrow(10true)/546,

nrow(11true)/546,

nrow(11true)/546)

##Counts number false positives per surveyor

1false <- 1found[(1found$3uid=='<NA>' & 1found$earliest=='NA' & 1found$latest=='NA'),]

nrow(1false)

3false <- 3found[(3found$3uid=='<NA>' & 3found$earliest=='NA' & 3found$latest=='NA'),]

nrow(3false)

2false <- 2found[(3found$3uid=='<NA>' & 2found$earliest=='NA' & 2found$latest=='NA'),]

nrow(2false)

4false <- 4found[(4found$3uid=='<NA>' & 4found$earliest=='NA' & 4found$latest=='NA'),]

nrow(4false)

5false <- 5found[(5found$3uid=='<NA>' & 5found$earliest=='NA' & 5found$latest=='NA'),]

nrow(5false)

6false <- 6found[(7found$3uid=='<NA>' & 6found$earliest=='NA' & 6found$latest=='NA'),]

nrow(6false)

7false <- 7found[(7found$3uid=='<NA>' & 7found$earliest=='NA' & 7found$latest=='NA'),]

nrow(7false)

8false <- 8found[(8found$3uid=='<NA>' & 8found$earliest=='NA' & 8found$latest=='NA'),]

nrow(8false)

9false <- 9found[(9found$3uid=='<NA>' & 9found$earliest=='NA' & 9found$latest=='NA'),]

nrow(9false)

10false <- 10found[(10found$3uid=='<NA>' & 10found$earliest=='NA' & 10found$latest=='NA'),]

nrow(10false)

11false <- 11found[(11found$3uid=='<NA>' & 11found$earliest=='NA' & 11found$latest=='NA'),]

nrow(11false)

11false <- 11found[(11found$3uid=='<NA>' & 11found$earliest=='NA' & 11found$latest=='NA'),]

nrow(11false)

f.total <- c(nrow(1false),

nrow(3false),

nrow(2false),

nrow(4false),

nrow(5false),

nrow(6false),

nrow(7false),

nrow(8false),

nrow(9false),

nrow(10false),

nrow(11false),

nrow(11false))

##Determines % of total that is false positive

f.frac <- c(

nrow(1false)/nrow(1found),

nrow(3false)/nrow(3found),

nrow(2false)/nrow(2found),

nrow(4false)/nrow(4found),

nrow(5false)/nrow(5found),

nrow(6false)/nrow(6found),

nrow(7false)/nrow(7found),

nrow(8false)/nrow(8found),

nrow(9false)/nrow(9found),

nrow(10false)/nrow(10found),

nrow(11false)/nrow(11found),

nrow(11false)/nrow(11found))

##Compiling Data Frame

table <- data.frame("participant" = c("1", "3", "2", "4", "5", " 6", "7", "8", "9", "10", "11", "11"),

"education" = c("u", "u", "u", "u", "g", "g", "g", "g", "p", "p", "p", "p"),

"andes"=c("y", "n", "n", "y", "n", "y", "y", "y", "y", "n", "y", "y"),

"gis" = c("n", "y", "n", "n", "n", "y", "n", "n", "n", "y", "y", "n"),

"exp" = c("y", "n","n", "y", "y", "n", "n", "n","y","y","y","y"),

"su4ess" = (t.total),

"failure"= (546-t.total),

"t.frac" = c(t.frac),

"f.total" = c(f.total),

"f.frac" = c(f.frac),

"time" = c(93, 71, 86, 103, 41, 51, 68, 99, 243, 99, 130, 65))

### Converting to binary

cnt2bin <- function(data, suc, fail) {

xvars <- names(data)[names(data)!=suc & names(data)!=fail]

list <- lapply(xvars, function(z) with(data, rep(get(z), get(suc)+get(fail))))

names(list) <- xvars

df <- as.data.frame(list)

with(data,data.frame(bin=rep(rep(c(1,0),nrow(data)),c(rbind(get(suc),get(fail)))),

df))

}

binarydat <- cnt2bin(table, "su4ess", "failure")

## Importing participant temporal data

1temp <- read.csv('')

2temp <- read.csv('')

3temp <- read.csv('')

4temp <- read.csv('')

5temp <- read.csv('')

6temp <- read.csv('')

7temp <- read.csv('')

8temp <- read.csv('')

9temp <- read.csv('')

10temp <- read.csv('')

11temp <- read.csv('')

11temp <- read.csv('')

## Assigning participants numeric values 1-12, 1 - 11, since glm doesn't like factors

# Creating dataframe for temporal analysis

tempdf <- rbind(1temp, 2temp, 3temp, 4temp, 5temp, 6temp, 7temp, 8temp, 9temp, 10temp, 11temp, 11temp)

11ite.csv(tempdf, ')

tempdf <- read.csv(')

class(tempdf)

# Model that doesn't take into a4ount random effec6

fit<-glm(tempdf$found~tempdf$latest+tempdf$surveyor, family=binomial())

summary(fit)

fit

# Model that DOES take into a4ount random effec6

glmerfit <- glmer(tempdf$found~tempdf$latest+(1|tempdf$surveyor)+(1|tempdf$3uid), family=binomial)

glmerfit

summary(glmerfit)

# Model that looks at site type

typesite <- read.csv('')

typeglmer <- glmer(data = typesite,found~type+(1|surveyor)+(1|3uid),family=binomial)

summary(typeglmer)

## Histogram of Proportion of Arch. Sites Found

hist(table$t.frac, col = 'grey', xlim = c(0,0.5), breaks = 10, probability = T, main = 'Verified Archaeological Sites Found', xlab = 'Proportion of Sites')

summary(table$t.frac)

t <- fitdistr(table$t.frac, "beta", start = data.frame(shape1 = 1.8837, shape2 =25.982)) ##Alpha and Beta calculated by hand

alpha <- t$estimate[1]

beta <- t$estimate[2]

t.x <- seq(0,1, length.out = 100)

t.y <- dbeta(t.x, shape1 = t$estimate[1], shape2 = t$estimate[2])

lines(t.y~t.x, col = 'red')

ks.test(table$t.frac, "pbeta", t$estimate[1],t$estimate[2])

t

meanagg = alpha/(alpha+beta)

sdagg = sqrt(alpha*beta)/((alpha+beta)^2*(alpha+beta+1))

meanagg

sdagg

## Histogram of Proportion of False Positives

hist(table$f.frac, col = 'grey', xlim = c(0,1), breaks = 10, probability = T, main = 'False Positives Recorded as Sites', xlab = 'Proportion of Sites')

summary(table$f.frac)

q <- fitdistr(c(f.frac), "beta", start = data.frame(shape1 = 1, shape2 =2)) ##Alpha and Beta calculated by hand

falpha <- q$estimate[1]

fbeta <- q$estimate[2]

q.x <- seq(0,1, length.out = 100)

q.y <- dbeta(q.x, shape1 = q$estimate[1], shape2 = q$estimate[2])

lines(q.y~q.x, col = 'red')

ks.test(table$f.frac, "pbeta", q$estimate[1],q$estimate[2])

fmeanagg = falpha/(falpha+fbeta)

fsdagg = sqrt(falpha*fbeta)/((falpha+fbeta)^2*(falpha+fbeta+1))

## Average False

mean(table$f.frac)

## boxplot levels of education for true found

educationexperience <- factor(table$education, levels = c('u', 'g', 'p'), ordered = T)

plot(table$t.frac~educationexperience, xlab = 'Level of Education', ylab = 'Sites Found', col = c('yellow', 'orange', 'red'), main = 'Proportion of Site Discovery Su4ess by Education')

## boxplot levels of education for false found

educationexperience <- factor(table$education, levels = c('Undergrad', 'Grad', 'Phd'))

plot(table$f.frac~educationexperience, xlab = 'Level of Education', ylab = 'False Positives', col = c('yellow', 'orange', 'red'), main = 'Proportion False Positives in Features Marked')

##boxplot graph comparing total true found andes

plot(table$t.frac~table$andes, xlab = 'Andean Specialist?', ylab = 'Proportion of Sites Found', col = c('red', 'blue'), main = 'Total Site Discovery Su4ess by Regional Specialization')

##boxplot graph comparing proportion false found andes

plot(table$f.frac~table$andes, xlab = 'Andean Specialist?', ylab = 'False Positives', col = c('red', 'blue'), main = 'Proportion False Positives in Features Marked')

##boxplot graph comparing proportion true found pedestrian survey experience

plot(table$t.frac~table$exp, xlab = 'Experience with Pedestrian Surveying?', col = c('red', 'blue'), ylab = 'Sites Found', main = 'Site Discovery Su4ess by Pedestrian Experience')

plot(table$f.frac~table$exp, xlab = 'Experience with Pedestrian Surveying?', col = c('red', 'blue'), ylab = 'False Positives', main = 'Proportion False Positives in Features Marked')

##boxplot graph comparing number true found by gis experience

plot(table$t.frac~table$gis, xlab = 'GIS Experience?', ylab = 'Sites Found', col = c('red', 'blue'), main = 'Site Discovery Su4ess by GIS Experience')

plot(table$f.frac~table$gis, xlab = 'GIS Experience?', ylab = 'False Positives Found', col = c('red', 'blue'), main = 'Proportion False Positives in Features Marked')

## Number of Archaeological Sites Discovered vs Time Spent Surveying in Minutes

plot(table$t.total~s$time..minutes., main = 'Sites Found vs Time', col = 'blue', cex = 1, pch = 16, xlab = 'Time Spent Surveying (Minutes)', ylab = 'Number of Sites Fonud')

##binomial regression categorical

head(binarydat)

binarytrue.actual <- glmer(bin ~ factor(education, ordered = T) + factor(andes) + factor(gis) + factor(exp) + time + (1 | participant) + (1|site), family = binomial(),data = binarydat)

summary(binarytrue.actual)summary(binarytrue)

found.predict <- predict(binarytrue,newdata=list(education="u", andes="n",gis="n",exp="n"),type='response',se.fit=TRUE,interval='confidence')

found.predict

##poisson regression categorical, false positive

poiss2 <- glm(f.total~factor(education, ordered=T)+factor(andes)+factor(gis)+factor(exp),data=table,family=poisson())

summary(poiss2)

falsepredict <- predict(poiss2, newdata=list(education='u',andes='n',gis='n',exp='n'), type='response')

falsepredict

## Composite True

par(mfrow=c(3,2))

educationexperience <- factor(table$education, levels = c('u', 'g', 'p'))

plot(table$t.frac~educationexperience, xlab = 'Level of Education', ylab = 'Proportion of Sites Found', col = c('yellow', 'orange', 'red'), main = 'Site Discovery Su4ess by Education')

plot(table$t.frac~table$andes, xlab = 'Andean Specialist?', ylab = 'Proportion of Sites Found', col = c('red', 'blue'), main = 'Site Discovery Su4ess by Regional Specialization')

plot(table$t.frac~table$exp, xlab = 'Experience with Pedestrian Surveying?', col = c('red', 'blue'), ylab = 'Proportion of Sites Found', main = 'Site Discovery Su4ess by Pedestrian Experience')

plot(table$t.frac~table$gis, xlab = 'GIS Experience?', ylab = 'Proportion of Sites Found', col = c('red', 'blue'), main = 'Site Discovery Su4ess by GIS Experience')

plot(table$t.frac~table$time, col = 'blue', cex = 1, pch = 16, xlab = 'Time Spent Surveying (Minutes)', ylab = 'Number of Sites Fonud', main = 'Number of Sites Found vs. Time')

hist(table$t.frac, col = 'grey', xlim = c(0,0.5), breaks = 10, probability = T, main = 'Verified Archaeological Sites Found', xlab = 'Proportion of Sites')

t <- fitdistr(table$t.frac, "beta", start = data.frame(shape1 = 1.8837, shape2 =25.982)) ##Alpha and Beta calculated by hand

alpha <- t$estimate[1]

beta <- t$estimate[2]

t.x <- seq(0,1, length.out = 100)

t.y <- dbeta(t.x, shape1 = t$estimate[1], shape2 = t$estimate[2])

lines(t.y~t.x, col = 'red')

par(mfrow=c(1,1))

##Composite False

par(mfrow=c(3,2))

plot(table$f.frac~educationexperience, xlab = 'Level of Education', ylab = 'False Positives', col = c('yellow', 'orange', 'red'), main = 'Proportion False Positives in Features Marked')

plot(table$f.frac~table$andes, xlab = 'Andean Specialist?', ylab = 'False Positives', col = c('red', 'blue'), main = 'Proportion False Positives in Features Marked')

plot(table$f.frac~table$exp, xlab = 'Experience with Pedestrian Surveying?', col = c('red', 'blue'), ylab = 'False Positives', main = 'Proportion False Positives in Features Marked')

plot(table$f.frac~table$gis, xlab = 'GIS Experience?', ylab = 'False Positives Found', col = c('red', 'blue'), main = 'Proportion False Positives in Features Marked')

plot(table$f.frac~table$time, col = 'blue', cex = 1, pch = 16, xlab = 'Time Spent Surveying (Minutes)', ylab = 'Number of False Positives', main = 'False Positives vs Time')

hist(table$f.frac, col = 'grey', xlim = c(0,1), breaks = 10, probability = T, main = 'False Positives Recorded as Sites', xlab = 'Proportion of Sites')

q <- fitdistr(table$f.frac, "beta", start = data.frame(shape1 = 0.5, shape2 =10)) ##Alpha and Beta calculated by hand

falpha <- q$estimate[1]

fbeta <- q$estimate[2]

q.x <- seq(0,1, length.out = 100)

q.y <- dbeta(q.x, shape1 = q$estimate[1], shape2 = q$estimate[2])

lines(q.y~q.x, col = 'red')

par(mfrow=c(1,1))
