# Supplementary material for: Unstructured satellite survey detects up to 20% of archaeological sites in coastal valleys of southern Peru
Source: PLoS One. 2024 Feb 6;19(2):e0292272. doi: 10.1371/journal.pone.0292272 (PMC10846729; doi:10.1371/journal.pone.0292272)
Supplement: Attachment — Submitted filename: Response to Reviewers.docx [file pone.0292272.s006.docx]

**Detailed Response to Reviewer Comments**

**Note:** Page and line numbers given by reviewers refer to the initial submission. Page and line numbers provided in the author responses refer to the page and line numbering in the revised document.

**R1:** *I should begin by noting that I have reviewed this manuscript twice before for another journal. I continue to see the issue of assessing success rates of unstructured satellite survey as of fundamental interest to many archaeologists, and the findings are more clearly presented than they initially were. However, in my read the paper still relies too much on quantitative analysis of a small dataset that is asked to support quite a lot. The quantitative analyses are undermined by the small sample size, making it a mistake in my opinion to focus on them. Rather, I would suggest minimizing the attempt to squeeze additional interpretations out of these data, and focusing instead on the central finding that detection rates are quite low.*

**Author Response:** Reviewer One’s concern about our small and heterogenous sample size is valid, and we take steps to follow their advice by shifting the focus away from the specific quantitative results of our findings and towards the broader finding that detection rates are quite low (page 19, lines 501 to 506; pages 21 & 22, lines 564 to 574; page 22 lines 580 to 581). We furthermore attempt to highlight that the method presented here can be applied to other regional contexts to derive detection rates specific to different conditions.

Additionally, we add a brief discussion of potential issues with a small and heterogenous sample size in our results section. In this addition, we provide our rationale for quantifying our models’ predictions and highlight that the impact of small sample sizes on accurate quantification of effect size for GLMM’s remains poorly understood (page 15, lines 415 to 422).

**R1:** *In fact, I think even rough approximations of detection and false positive rates from unstructured satellite survey are quite valuable. Even those generalizations, however, are built on a sample of only twelve. Focusing on the categorical separation of those twelve into three types with only four samples each puts even the most rigorous quantitative analysis on shaky ground. Do the groups of participants constitute random and/or representative samples of undergrads/grads/PhDs? With respect to which particular aspects of those populations? Are the differences between the groups large enough – given the small sample sizes – to constitute compelling evidence about the populations in question? The variances in sites found and false positives in each group suggest that these small samples are *very* heterogenous and thus likely to not characterize the populations (of undergrads/grads/PhDs) very well.*

**Author Response:** Reviewer One’s concern about our small and heterogenous sample size is valid, and we take steps to follow their advice by shifting the focus away from the specific quantitative results of our findings and towards the broader finding that detection rates are quite low (pages 22 and 23, lines 590 to 604). Nonetheless, the relationship between experience and detection rate is qualitatively evident in the boxplots, statistically robust as indicated by the GLM output, and theoretically sound (i.e. it shouldn’t be surprising that training affects success rate). We therefore maintain that our analysis is identifying meaningful statistical relationships.

**R1:** *I noted something similar in a previous review and I think this comment (lightly edited here) remains applicable to the current version:
To my mind, the overall results have interesting and useful things to say about likely success rates of imagery-based survey (even under what are, I would judge, effectively pretty optimal conditions on the S. Coast of Peru). That central message, to me, is a much more important (and robust) result than the attempts to identify characteristics of participants that affect their success rates. With respect to the latter, sample sizes are quite small (4 each of undergrad, grad, and PhD students), and no attempt is made to argue that the samples are in any way representative (nor do I imagine that such an argument would be possible). To me this suggests that some re-emphasis make for a more compelling paper, with a central message something like, “results may vary by experience and expertise, but even under optimal conditions (advanced education, experience in the region and with both GIS and pedestrian survey) satellite survey only identifies ~20% of sites”. That is, the variables considered apparently can matter, but even in the best-case scenario identification rates are quite low.*

**Author Response**: We ultimately agree with Reviewer One that the primary focus of the paper ought to be the relative ineffectiveness of satellite survey using freely available satellite imagery compared to traditional archaeological survey. However, we maintain that our statistical analyses of the extent to which different levels and types of experience can impact the success of satellite survey have value. To incorporate both of these perspectives we add a hedging language throughout our conclusion, highlighting the limits of our models without removing them from the article. In particular, we add language noting that our particular quantitative results should only be utilized in southern coastal Peru (page 22, lines 580 to 581).

**R1:** *The last two paragraphs of the intro read more like a grant proposal than an article, to me. I’d suggest that here the focus should be on what the *findings* are, rather than what the hypotheses are.*

**Author Response**: We respectfully disagree with the reviewer regarding the role of the introduction in a scientific article. In our view, the role of the introduction should be to contextualize the purpose of the study, explain the research question, and present hypotheses. We fear that by focusing on the findings of our research in the introduction, we may bias readers in their interpretation of our results before they consider our methodology. Nonetheless, the abstract foreshadows findings, which are presented more fully in the results section.

**R1:** *There are several instances of “Error! Bookmark not defined.” codes appearing in the text (p4, 6, 13)*

**Author Response:** These errors have been resolved.

**R1**: “*Further hampering all satellite survey approaches is the fact that features of archaeological sites that may be visible via satellite imagery may be obscured by modern architecture and therefore invisible to satellite photographs without field observations, excavation, or ground penetrating radar (33).”

I find this confusingly written, and would strike out the clause “that may be visible via satellite imagery”. I’d also suggest replacing “modern architecture” with the broader “modern development”.*

**Author Response**: We follow Reviewer One’s advice and incorporate their suggestions.

**R1:** *p7
“While there are no pre-existing models to suggest the proportion of archaeological sites that can be found through unstructured satellite survey, clearly the proportion must lie between 0–100%. We further expect the proportion to be on the low end of this range”

I would strike out “clearly the proportion must lie between 0–100%”, since it’s stating the obvious. The following sentence could be simplified to just, “We expect the proportion to be low”.*

**Author Response**: We follow Reviewer One’s advice and incorporate their suggestions.

**R1:** *p8
“Third, the lack of dense surface vegetation is ideal for remote detection by satellite imagery.”

Should be reworded to something like, “lack of dense surface vegetation provides ideal conditions for remote detection”.*

**Author Response:** We follow Reviewer One’s advice and incorporate their suggestions.

**R1:** p9 Section on “Satellite Survey”

This whole section should not be in the present tense. i.e., “were recruited to perform” rather than, “are recruited to perform”.

**Author Response:** We correct this error.

**R1:** *p16
“Our binomial GLMM results demonstrate that the following variables significantly (p < 0.1) affect site detection rates: educational experience, experience in Andean archaeology, experience working with GIS, and time spent surveying”

These variables are not independent of one another: experience in Andean archaeology and working with GIS are likely to be correlated with educational experience.*

**Author Response:** Although strictly independent variables are ideal for generalized linear models, there are no requirements that the variables in a GLMM like ours need to be strictly independent—so long as they are non-redundant. However, we acknowledge that we call these variables independent in our original draft and correct this error (page 13, line 382). While it would be technically possible to model these variables as interacting, we believe such an approach would be irresponsible given the limitations of our sample size.

**R1:** p19
*I’m not sure I follow the distinction between site age and site type that’s used here. The site types listed are from time periods that are (to an extent) distinct.*

**Author Response**: Our logic here is that it is conceivable that that an older archaeological culture may be more visible to satellite survey than a younger one depending on traits such as agricultural and architectural practices. Thus, we observe both site type and site age as there is *some* overlap–and that by looking at both we are able to assess the relative impact of time and cultural practices on visibility to satellite surveyors.

**R1:** *p20, Ln 375
“site discovery rates are likely to be much higher than in unstructured surveys”

Should this read “structured surveys”?*

**Author Response**: Yes, we edit this line to improve the clarity of our statement (page 19, line 476).

**R1:** *Table 2
Row 1, ‘Sites Found’ column reads “25 (4%)”, but should read “25 (.04)” to match the other rows.*

**Author Response**: We correct this error.

**R1:** *Figs. 3 and 4:
Some other color than red should be used for the ‘p’ boxplot in (A), to avoid the impression of comparable coding with B, C, and D. In fact, it’s not clear that color is adding anything to these boxplots at all. All of them should also specify the samples sizes.
For (E) in both figures, either the y-axis tick labels or the y-axis is mislabeled. For (F), these appear to be showing frequency rather than density and the y-axis should reflect that.*

**Author Response**: Color is only included in these plots to allow potential readers to quickly differentiate between groups at a glance. The color scale used in boxplot A has been changed to avoid the impression of comparable coding with B, C, and D. Sample size has been added to the caption for Figures 2 and 3. Additionally, we make the recommended changes to boxplots E and F. Sample sizes for each subset tested are presented in Table 2; we choose not to replicate that information here to avoid over-complicating an already busy figure.

**R2:** *Image data: No information about the image data is provided, even though it is a crucial factor in the analysis. While commercial portals such as the ones used provide no detailed data, they do provide some basic data, e.g. in Google Earth/Maps the satellite/sensor used and the year of acquisition. This gives some indication of image properties and quality, which may vary greatly across the study areas and thus affect the results. The second parameter is important as it defines the time passed between the archaeological survey and the time the images were acquired, during which the situation on the ground might have changed.*

**Author Response**: Unfortunately, Microsoft does not provide information regarding the satellite/sensor used and the year of acquisition for Bing Maps for free. As this study was done without specific grant funding, we are unable to gain access to this information. While Google Earth/Maps *does* report the sensor used and the date of acquisition, the quality of the images are poor for the study area in question. Thus, we made a tradeoff and decided to focus user the higher-quality but information poor Bing Maps imagery. We note this shortcoming of our research in the Methods section (page 10 , lines 299 to 301).

**R2:** *Archaeological data: The site datasets are generally well chosen but do not represent the full archaeological record of the study areas, which the authors fails to discuss. The survey projects probably missed some sites while others were lost since. Furthermore, the sites probably vary greatly in type, size, preservation etc., but this is not discussed either even though it is another important parameter for the task in question.*

**Author Response:** While we do not cover the entirety of the full archaeological record of the study areas, we believe that our comparisons to specific boots-on-the-ground archaeological survey and satellite surveillance have merit. However, following from Reviewer Two’s suggestions we make the limitations of the data we are able to draw from in the context of this project clear in the methods section (page 11, lines 323 to 331).

**R2:** *Finally, a number of minor editorial issues that should be addressed:
50: in the Yucatan => in Yucatan
82: the process site discovery => the process of site discovery
99: to search successfully search => to successfully search
219: not reported for the Goldstein 2005, but => not reported for the Goldstein (2005) survey, but
Table 2, participant 1: (4%) => (0.04)
424: factors might be considered in future research might include => factors to be considered in future research might include
434: other variables such as seasonal effects such as vegetation changes => other variables such as seasonal effects (e.g., vegetation changes)
475: Journal of archaeological science => Journal of Archaeological Science
488: International journal of applied earth observation and geoinformation => International Journal of Applied Earth Observation and Geoinformation
522: Incomplete reference
558: Journal of archaeological Science => Journal of Archaeological Science
580: In: th Annual Meeting => add number
Fig. 3E: Number of sites found => Proportion of sites found
Fig. 4E: Number of false positives => Proportion of false positives
Supporting information 2, column B heading: Eating => Easting
Supporting information 4, column C heading: Participant => Northing*

**Author Response:** We address and correct all of the listed changes.
